# Vacancy-dependent stability of cubic and wurtzite Ti_1−*x*_Al*_x_*N

**DOI:** 10.1016/j.surfcoat.2015.05.017

**Published:** 2015-08-15

**Authors:** H. Euchner, P.H. Mayrhofer

**Affiliations:** Institute of Materials Science and Technology, Technische Universität Wien, 1060 Vienna, Austria

**Keywords:** Ti_1−*x*_Al*_x_*N, Vacancies, Density functional theory

## Abstract

While it is well-known that supersaturated cubic-structured Ti_1−*x*_Al*_x_*N can be prepared by physical vapor deposition, the impact of point defects on formation process and cubic to wurtzite transition is largely unexplored. Irrespective of point defects, ab initio calculations correctly predict the Al concentration of the cubic to wurtzite transition. By means of density functional theory we show that vacancies on metal and/or non-metal sites only slightly affect the cubic to wurtzite transition region, whereas they clearly affect the physical properties.

## Highlights

• No significant impact of vacancies on the cubic to wurtzite transition in Ti_1−*x*_Al*_x_*N
• Explanation of agreement of experiment and simulation despite negligence of vacancies
• Impact of vacancies on phase stability of metastable Ti_1−*x*_Al*_x_*N
• Influence of vacancies on lattice parameter and elastic properties in Ti_1−*x*_Al*_x_*N

## 1 Introduction

Industrial applications have a large demand for protective coatings with high hardness, good elastic properties and improved ductility. With respect to this requirement profile, transition metal (TM) nitrides, especially the cubic-structured Ti_1−*x*_Al*_x_*N and closely related phases, have proven to be of great interest [1,2]. The cubic Ti_1−*x*_Al*_x_*N phase is well known to be meta-stable with respect to decomposition into cubic (rocksalt) TiN and hexagonal (wurtzite) AlN [3,4]. The fact that cubic-structured Ti_1−*x*_Al*_x_*N, despite its meta-stability, can be synthesized in thin film coatings has numerous advantages. First, the cubic phase has superior physical properties, like hardness or elastic moduli as compared to its wurtzite counterpart. Additionally, the self-hardening effect at higher temperatures – resulting from the spinodal decomposition into cubic AlN and cubic TiN – makes Ti_1−*x*_Al*_x_*N well-suited for applications at elevated temperatures. Moreover, passivation due to the formation of Ti and Al oxides at higher temperatures creates corrosion barriers. These unique properties of super-saturated Ti_1−*x*_Al*_x_*N result from the interplay and competition between two phases that prefer different structure types — cubic TiN and wurtzite AlN. It has been shown that cubic-structured Ti_1−*x*_Al*_x_*N thin films with Al contents of up to 70% can be grown by physical vapor deposition (PVD), while at higher Al contents the wurtzite phase becomes the preferred structure type. Ab initio studies on the phase stability of cubic-structured nitrides, such as Ti_1−*x*_Al*_x_*N, Zr_1−*x*_Al*_x_*N or Hf_1−*x*_Al*_x_*N [5], perfectly reproduce the cubic to wurtzite transition evidenced in PVD studies. However, PVD methods, such as reactive sputtering, are known to result in an incorporation of point defects during the growth process. Recently, it was shown that in case of Ti_1−*x*_Al*_x_*N vacancies are the dominant defect type [6], while interstitials are energetically less favorable. While the influence of vacancies on the decomposition pattern [7] of cubic-structured Ti_1−*x*_Al*_x_*N has been investigated, the cubic to wurtzite transition has so far only been studied for its pressure dependence [8]. The impact of vacancies on this transition, however, has never been investigated. Thus the question arises, why ab initio calculations, completely neglecting the presence of point defects, are able to excellently predict the stability regime of ternary nitrides. To address the question, how vacancies actually influence growth kinetics and phase stability, we have investigated solid solutions of cubic- and wurtzite-structured Ti_1−*x*_Al*_x_*N with different vacancy contents on metal and nitrogen sublattice, respectively. Somewhat surprising, the impact of vacancies is almost negligible and the Al concentration at the transition remains basically unchanged. However, it has to be pointed out that, while cubic-structured Ti_1−*x*_Al*_x_*N easily accommodates vacancies, wurtzite-type Ti_1−*x*_Al*_x_*N has proven to be quite sensitive to vacancies, resulting in much stronger distortions of the local environment. Moreover, it could be shown that in case of cubic-structured Ti_1−*x*_Al*_x_*N, metal vacancies result in a decreasing lattice parameter, whereas nitrogen vacancies leave the lattice parameter almost unaffected. Consequently, this study also justifies earlier results, which are obtained for defect free ternary nitrides. Here, it has to be pointed out that Ti_1−*x*_Al*_x_*N seems to be a very fortunate case, meaning that in other material systems the impact of vacancies may be much stronger. One such example is the formation of AlB_2_-structured WB_2_. Whereas this crystal structure so far could not be obtained for bulk material [9], it was recently successfully deposited with PVD [10]. For this system, computational studies indicate that the formation of AlB_2_-structured WB_2_ in PVD processes may be explained by an increased vacancy content [11], thus clearly evidencing the strong impact vacancies may have.

## 2 Computational methods

Structure and stability of supersaturated Ti_1−*x*_Al*_x_*N solid solutions were studied by ab initio simulations, applying the density functional theory (DFT) code VASP (Vienna Ab Initio Simulation Package) [12–14]. Using the projector augmented waves method within the generalized gradient approximation (PAW-GGA), supercells of cubic- and wurtzite-structured Ti_1−*x*_Al*_x_*N were investigated. Three different cases were considered: perfect structures, vacancies on the metal sublattice and vacancies on the nitrogen sublattice. The respective defect free structures were created via the special quasi-random structures (SQS) approach [15], using the atat package [16]. Applying this approach, different Ti/Al concentrations were realized in defect free supercells of 32 and 64 atoms. These wurtzite- and cubic-structured supercells were then optimized with respect to atomic position and lattice parameter using a 4 × 4 × 8 k-point-mesh for the cubic 32 atom structure, whereas a 6 × 6 × 4 mesh was used for the 32 atom wurtzite cells. The k-point meshes of the 64 atom cells, were adjusted such that the k-point resolutions remained the same as in the 32 atom case. Moreover, all calculations were conducted with an energy cutoff of 700 eV. In a next step, vacancies were created by removing either an aluminum or a nitrogen atom. The introduction of a metal/nitrogen vacancy in a 64 atom supercell results in a vacancy concentration of 3.125% on the respective sublattice, while for a 32 atom supercell the vacancy concentration corresponds to 6.25%. After removing a selected metal/nitrogen atom, atomic position and lattice parameter of the vacancy containing structures were again optimized, using the same settings as for the defect-free structures. In case of introducing a metal vacancy, one has to keep in mind that this results in a changed Ti to Al ratio and thus a shift in chemical composition of the metal sublattice. To investigate the influence of the exact position of the vacancy in the 32 atom supercells, we have repeated the calculations for several different vacancy configurations at each composition. In the wider vicinity of the cubic to wurtzite transition we have investigated all possible vacancy configurations to rule out effects due to a specific choice.

## 3 Results and discussion

To determine the effect of the different chemical and vacancy concentrations, the energy of formation, *E_f_*, of both structural modifications of Ti_1−*x*_Al*_x_*N was determined, following Eq. (1):

(1) $$E_{f}=\frac{1}{\sum_{i}n_{i}}\left(E_{\mathrm{tot}}-\sum_{i}n_{i}E_{i}\right)$$

with *E_tot_* and *E_i_* the total energy of the compound and its elemental constituents, as determined from DFT and *n_i_* the number of atoms of species *i*. Thus, in our case, the energy of formation describes the energy that is gained when a (Ti_1−*x*_Al*_x_*)_1−*y*_N_1−*z*_ alloy is formed from *α*-Ti, fcc-Al and molecular nitrogen.

**Fig. 1:**
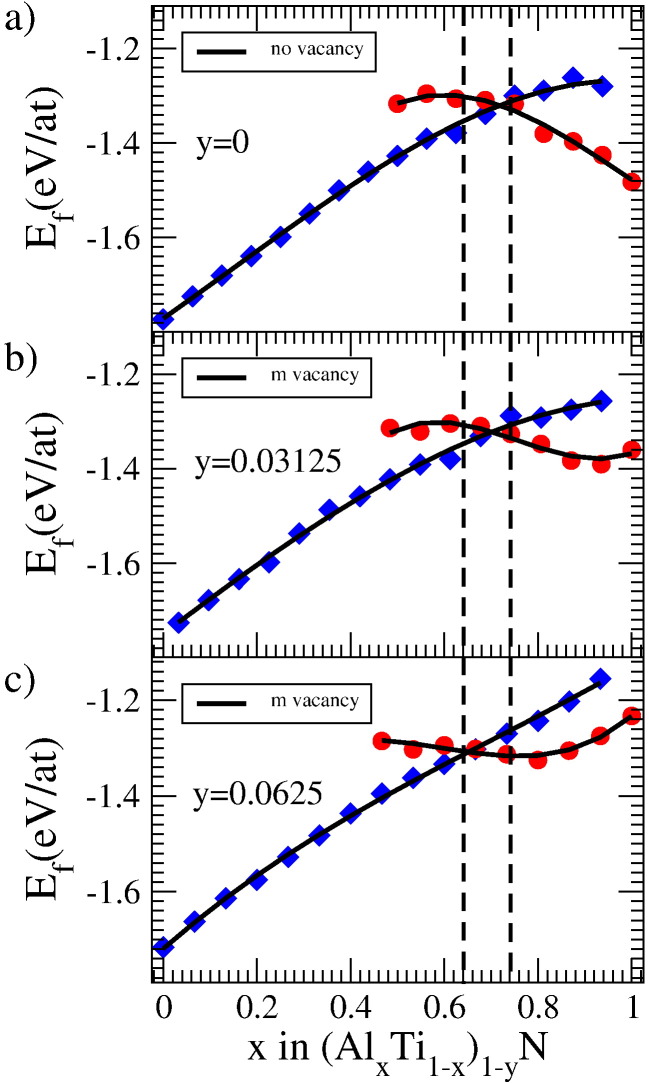
*E_f_* of defect free (a) and metal deficient (b, c) (Ti_1−*x*_Al*_x_*)_1−*y*_N. Results for 64 (b) and 32 (c) atom supercells with one vacancy on the metal sublattice are shown. The energy values for the 32 atom cell are obtained from averaging over different vacancy configurations for each composition. The error bars (here smaller than the symbol size) denote the standard deviation with respect to the different vacancy configurations. Black curves are third order polynomial fits to the data.

**Fig. 2:**
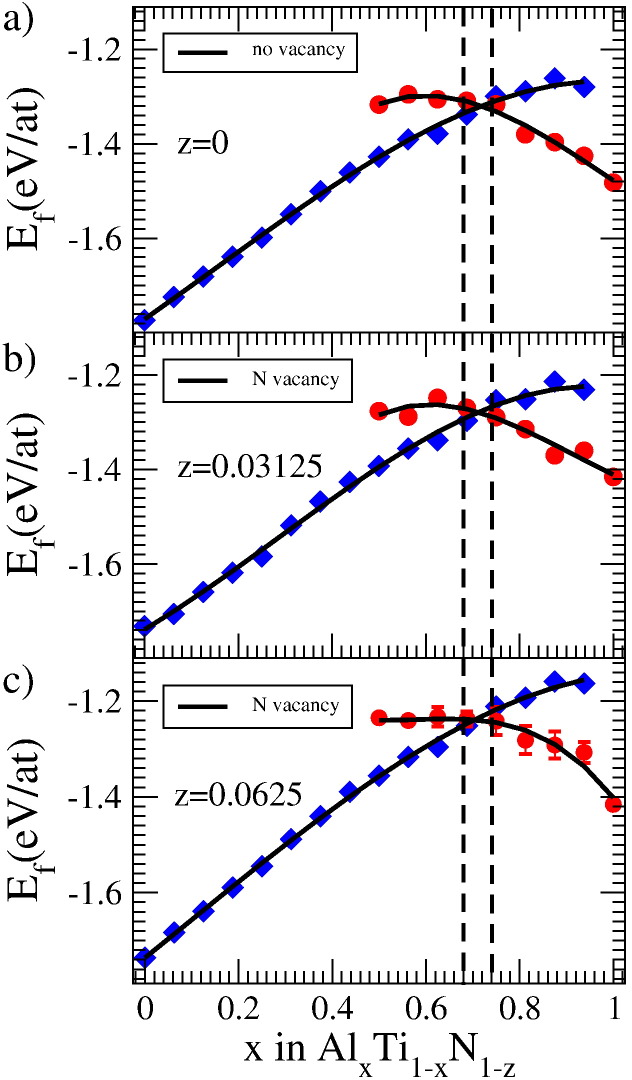
*E_f_* of defect free (a) and nitrogen deficient (b, c) Ti_1−*x*_Al*x*N_1−*z*_. Results for 64 (b) and 32 (c) atom supercells with one vacancy on the nitrogen sublattice are shown. The error bars in panel (c) are obtained as described in Fig. 1. Black curves are third order polynomial fits to the data.

Figs. 1a) and 2a) depict the energy of formation of defect free cubic- and wurtzite-structured Ti_1−*x*_Al*_x_*N with respect to its chemical composition. In agreement with previous reports [5], we find the transition from cubic to wurtzite structure at an Al concentration of about 70%.

Panels b) and c) of Fig. 1 show the energy of formation for structures containing vacancies on the metal sublattice, using a 32 and 64 atom-sized supercell, respectively. Indeed, the introduction of vacancies has a small but non negligible impact on the stability of cubic- and wurtzite-structured (Ti_1−*x*_Al*_x_*)_1−*y*_N. Whereas the transition concentration is almost unaffected for the 64 atom supercell, evidencing only a tiny shift towards lower Al concentrations, the smaller unit cell clearly shows the impact of the increased vacancy concentration (see Fig. 1c)). For this case, the vacancy on the metal sublattice results in a small but non negligible shift of the cubic to wurtzite transition towards lower Al concentrations. This can be seen from the dashed, vertical lines in Fig. 1, which roughly mark the upper and lower limit of the transition regime. Moreover, the shape of the energy curve of wurtzite (Ti_1−*x*_Al*_x_*)_1−*y*_N changes gradually from concave to convex when increasing the vacancy content, thus pointing to an incipient instability of the wurtzite phase.

The similar slopes of metal deficient cubic and wurtzite *E_f_* curves for Al contents above 0.7 suggest that the transition region will be extremely sensitive to additional effects, such as for instance internal stresses. With increasing compressive stresses the cubic phase is, due to the lower specific volume, preferred over its hexagonal counterpart, as could be shown in previous computational studies [17]. Consequently, compressive stresses may significantly shift the transition in metal deficient (Ti_1−*x*_Al*_x_*)_1−*y*_N towards higher Al contents.

The energy of formation for nitrogen deficient cells is depicted in Fig. 2b) and c). For both vacancy concentrations the cubic to wurtzite transition seems essentially unaffected. Due to the larger spread of the data, especially for the wurtzite structure, a slight shift is certainly possible. However, the width of the transition regime, marked by the vertical, dashed lines, is clearly smaller as compared to the structures containing metal vacancies. Furthermore, the energy curves of the nitrogen deficient wurtzite structure are clearly less affected by the increased vacancy content. Their overall shape remains similar for the different vacancy contents, whereas for the case of metal vacancies, significant shape changes were observed, as becomes evident from a comparison of Figs. 1 and 2.

The above discussion has its focus on the energy of formation, thus neglecting the impact of entropic contributions. This is justified, since the configurational entropy depends only on the chemical composition, such that it is equivalent for cubic- and wurtzite-structured Ti_1−*x*_Al*_x_*N. Vibrational entropy on the other hand may differ for both structural modifications, yet it will only yield significant contributions at elevated temperatures. Therefore it is valid to assume that the cross-over from cubic to wurtzite structure can accurately be determined from the energy of formation.

From the above discussed energies of formation of defected and defect-free Ti_1−*x*_Al*_x_*N, the formation energy of nitrogen vacancies can easily be determined as:

(2) $$E_{f}^{vac}\left[N\right]=\frac{n}{2}\left(E_{\mathrm{tot}}\left[Ti_{1-x}Al_{x}N\right]-E_{\mathrm{tot}}\left[Ti_{1-x}Al_{x}N_{1-z}\right]-z\frac{1}{2}E_{\mathrm{tot}}\left[N_{2}\right]\right)$$

with *n* being the total number of atoms, whereas *E*_*tot*_[*Ti*_1−*x*_*Al*_*x*_*N*] and *E*_*tot*_[*Ti*_1−*x*_*Al*_*x*_*N*_1−*z*_] denote the total energy of a formula unit of defect-free and defected Ti_1−*x*_Al*_x_*N, while *E*_*tot*_[*N*_2_] is the total energy of molecular nitrogen.

For metal vacancies the formalism becomes a bit more demanding since one has to account for a changing stoichiometry. The formation energy of a defect then takes the following appearance:

(3) $$E_{f}^{vac}\left[M\right]=\frac{n}{2}\left(E_{\mathrm{tot}}\left[Ti_{1-x}Al_{x}N\right]-E_{\mathrm{tot}}\left[{\left(Ti_{1-x}Al_{x}\right)}_{1-y}N\right]-y[\left(1-x\right)E_{\mathrm{tot}}\left[Ti\right]+xE_{\mathrm{tot}}\left[Al\right]]\right)$$

with *n* being the total number of atoms, whereas *E*_*tot*_[*Ti*_1−*x*_*Al*_*x*_*N*] and *E*_*tot*_[(*Ti*_1−*x*_*Al*_*x*_)_1−*y*_*N*] denote the total energy of a formula unit of defect-free and defected Ti_1−*x*_Al*_x_*N. Finally, *E*_*tot*_[*Ti*] and *E*_*tot*_[*Al*] represent the total energy of a pseudo-atom, consisting of *α*-Ti and fcc-Al weighted by their stoichiometric abundances. The introduction of this pseudo-atom ensures a non-changing stoichiometry when comparing deficient and non-deficient structure as it would be the case for the creation of a vacancy in a macroscopic system.

**Fig. 3:**
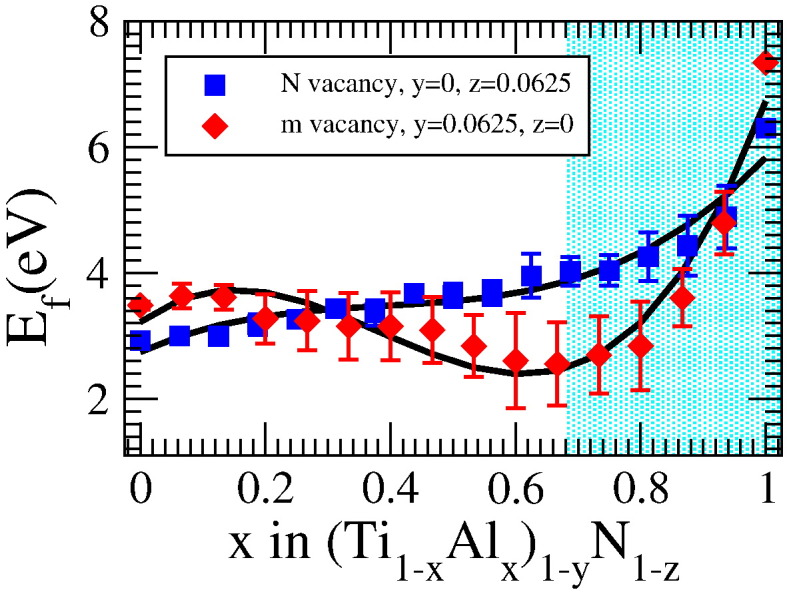
Vacancy formation energies for c-(Ti_1−*x*_Al*_x_*)_1−*y*_N_1−*z*_ with vacancies on metal (red diamonds) and nitrogen (blue squares) sublattice, depicted over the aluminum content, as obtained for the 32 atom supercells. In case of metal vacancies, the error bars result from the averaging over the different configurations, as well as from the interpolation of the stoichiometry of the perfect crystal. For nitrogen vacancies the error bars result only from the average over the different configurations. The shaded area indicates the region in which the wurtzite structure is already the more stable one.

Due to the limited supercell sizes in our calculations the stoichiometry on the metal sublattice of metal deficient and vacancy-free structures are slightly different. Therefore, to access the correct vacancy formation energies for the case of metal vacancies, we have interpolated the energies of the perfect crystal over the whole composition range, using third order polynomial fits. Thus the energy of perfect structures with the same stoichiometry on the metal sublattice as the metal-deficient structures could be extracted. The vacancy formation energies, corresponding to the 32 atom supercells and determined following Eqs. (2) and (3), are depicted in Fig. 3. For nitrogen vacancies it is clearly visible that the vacancy formation energy first increases almost linearly with the aluminum content. Starting slightly below 3 eV for pure c-TiN it mounts up to over 6 eV for pure c-AlN. For metal vacancies the dependence on the aluminum content is less evident. The vacancy formation energy is slightly decreasing, from about 3.5 eV to slightly above 2.5 eV, before it raises up to almost 7.5 eV for pure c-AlN. It is interesting to note that in both cases the vacancy formation energies are rather similar, thus pointing out that the formation of metal vacancies is not unlikely, as was also recently shown for the case of Ti_0.5_Al_0.5_N [6]. Moreover, when passing into the region where the cubic structure becomes less stable, a strong increase of the vacancy formation energy is evidenced for both metal and nitrogen vacancies.

**Fig. 4:**
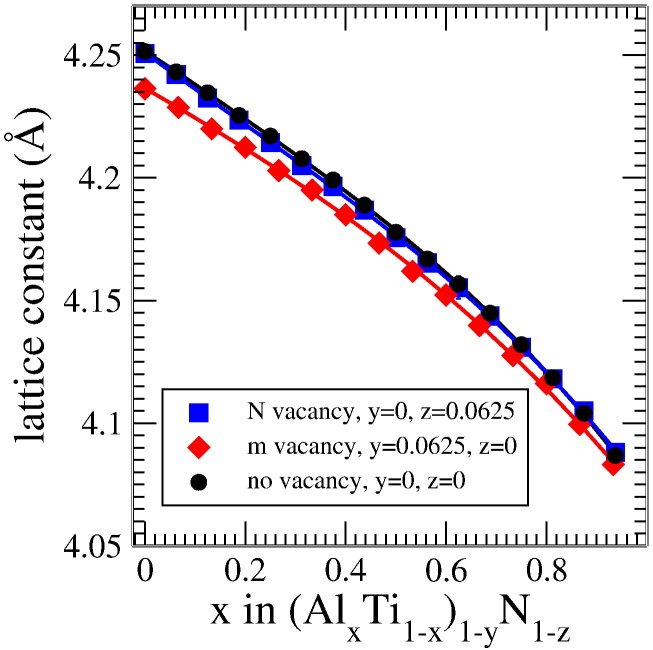
Lattice parameter of cubic (Ti_1−*x*_Al*_x_*)_1−*y*_N_1−*z*_ without vacancies (black circles), and with vacancies on metal (red diamonds) and nitrogen (blue squares) sublattice, depicted over the aluminum content, as obtained for the 32 atom supercells. The vacancy case is averaged over the different configurations, the error bars are smaller than the symbol size.

Apart from investigating the changes in energy, it is also elusive to study the evolution of the lattice parameter for different vacancy concentrations. In cubic-structured Ti_1−*x*_Al*_x_*N the introduction of nitrogen vacancies has a rather tiny effect on the lattice constant, meaning that only a slight decrease is evidenced (see Fig. 4), whereas the trend of a decreasing lattice constant with increasing Al concentration clearly remains unchanged. In case of vacancies on the metal sublattice, a significant decrease of the lattice parameter is visible.

With respect to the analysis of diffraction data this is an important outcome, since a peak shift towards larger 2*θ* angles may thus also be explained by nitrogen overstoichiometry, resulting from metal vacancies. In fact, cubic-structured Ti_1−*x*_Al*_x_*N easily accommodates vacancies, which is also reflected in the few relaxation steps necessary to balance a vacant site.

For wurtzite Ti_1−*x*_Al*_x_*N the story is rather different. The a-axis slightly decreases with increasing Al concentration and remains similar for defect free as well as nitrogen and metal deficient Ti_1−*x*_Al*_x_*N. For the c-axis much stronger changes are observed. The vacancy free structure shows a slight tendency versus a decrease of the lattice parameter with increasing Al content. When vacancies are introduced, the lattice parameter strongly fluctuates and for certain configurations deviates strongly from the defect free one. This results in some of the structures having a strongly decreased c/a ratio, which indicates, that the structure is approaching the so-called B*_k_* structure. Thus, the presence of vacancies may be seen as a driving force for stabilizing the B*_k_* structure with respect to wurtzite. These findings are not surprising, since it was already known that for lower Al concentrations the B*_k_* structure is more stable than wurtzite [5].

To investigate the impact of vacancies on the physical properties of cubic-structured Ti_1−*x*_Al*_x_*N, we have determined Young's, bulk and shear modulus at four different Al concentrations. These properties can directly be obtained from the single crystal elastic constants, which in turn can be determined by evaluation of the stress–strain relation, using the universal linear independent coupling strain approach [18]. Following this approach, the relaxed supercells were deformed by six linearly independent strains, resulting in six different strain states. The corresponding stresses were then extracted after optimization of the atomic positions of these six configurations, however, at fixed lattice constants. These structure optimizations of the strained configurations were again conducted using the VASP code, applying the same settings as in the above described stability calculations.

After extraction of the respective stresses, the elastic constants can be determined from the stress–strain relation by linear least square fits using single value decomposition [18]. Due to the elastic tensor being symmetric, it in principle contains 21 elements — the elastic constants. Yet, by directly imposing cubic symmetry to the elastic tensor, only six elastic constants remain to be determined from single value decomposition. To ensure the dynamical stability of the investigated cubic-structured solid solutions, the stability criterion for cubic crystals

(4) $$C_{11}>\left|C_{12}\right|,\;C_{44}\;>0,\;C_{11}+2C_{12}>0$$

need to be fulfilled. Indeed, all investigated compositions were dynamically stable. From the calculated elastic constants the polycrystalline average of Young's modulus (*Y*), bulk modulus (*B*), and shear modulus (*G*) was determined. While the so-called Voigt average, can be considered as the upper limit [19], its counterpart, the Reuss average, gives a lower bound [20]. For the bulk modulus of a cubic crystal both averages are identical [21]:

(5) $$B_{v}=B_{r}=\frac{1}{3\left(C_{11}+2C_{12}\right)}\text{.}$$

For the shear modulus, the two results have the following appearance [21]:

(6) $$G_{v}=\frac{1}{5}\left(C_{11}-C_{12}+3C_{44}\right)$$

(7) $$G_{r}=\frac{1}{\left(4/\left(5\left(C_{11}-C_{12}\right)\right)+3/(5C_{44})\right)}\text{.}$$

For a further discussion, concerning the impact of vacancies, the Voigt–Reuss–Hill average, *G*_*vrh*_ and *B*_*vrh*_ are investigated, with *G*_*vrh*_ = (*G*_*r*_ + *G*_*v*_)/2 and *B*_*vrh*_ = *B*_*r*_ = *B*_*v*_ [21]. Finally the Young's modulus in the Voigt–Reuss–Hill approximation is defined as [21]:

(8) $$Y_{vrh}=\left(9B_{vrh}G_{vrh}\right)/\left(3B_{vrh}+G_{vrh}\right).$$

**Fig. 5:**
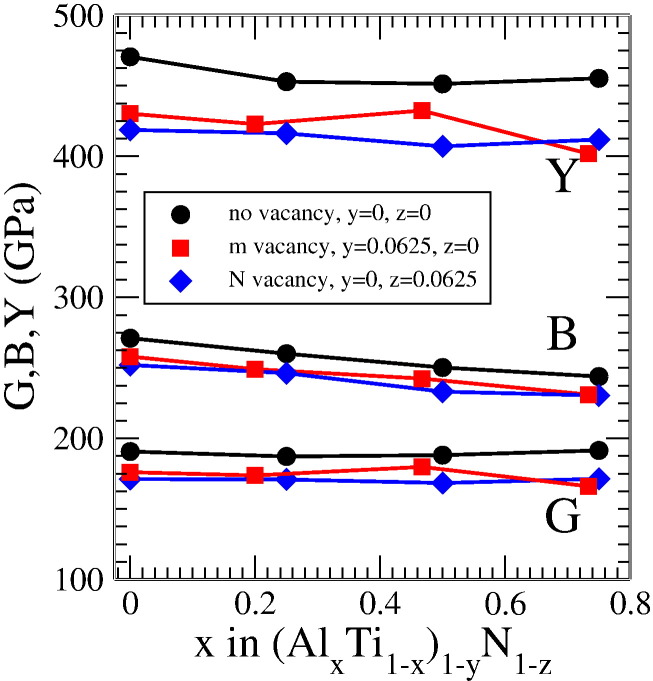
Young's (Y), bulk (B) and shear (G) modulus of cubic (Ti_1−*x*_Al*_x_*)_1−*y*_N_1−*z*_ without vacancies (black circles), and with vacancies on metal (red squares) and nitrogen (blue diamonds) sublattice, obtained for 32 atom supercells containing one vacancy.

*Y*_*vrh*_, *G*_*vrh*_ and *B*_*vrh*_ are depicted in Fig. 5 for both vacancies on the metal and the nitrogen sublattice. The resulting data are compared to defect free structures at four different Al concentrations. The results for defect-free Ti_0.5_Al_0.5_N (B = 250 GPa, G = 188 GPa, E = 451 GPa) as well as the general trends are in good agreement with previous works [22,23]. For vacancy free as well as N and metal deficient Ti_1−*x*_Al*_x_*N the same trends are evidenced. Indeed, an increasing Al content results in decreasing Young's and bulk modulus, whereas the shear modulus remains essentially constant. While *Y*_*vrh*_, *G*_*vrh*_ and *B*_*vrh*_ differ only slightly for nitrogen and metal deficient cubic-structured Ti_1−*x*_Al*_x_*N, they are clearly increased for vacancy free Ti_1−*x*_Al*_x_N*.

## 4 Conclusion

In this letter we have shown that Ti_1−*x*_Al*_x_*N, despite being a polar material, is not very sensitive to point defects. Indeed, the Al concentration at which the cubic to wurtzite transition in Ti_1−*x*_Al*_x_*N takes place is only slightly affected by the presence of point defects. For a vacancy concentration of 3.125% on the metal sublattice, we have evidenced a tiny shift of the transition towards a lower Al content. This tendency is more pronounced with higher vacancy concentration, however, the changes are most likely too small to be validated experimentally. The transition of nitrogen deficient cells on the other hand seems to be insensitive to the presence of vacancies. Even for a vacancy concentration of 6.25% on the nitrogen sublattice the transition concentration is essentially unaffected. The vacancy formation energies of metal and nitrogen vacancies in cubic Ti_1−*x*_Al*_x_*N are rather similar. For aluminum concentrations of less than 70% they are found to lie roughly between 2.5 and 4 eV. For higher aluminum contents, i.e. in the region where the wurtzite phase is already more stable, the vacancy formation energy in c-Ti_1−*x*_Al*_x_*N increases strongly for both vacancy types. Due to PVD deposited materials being full of point defects, similar to materials exposed to extremal radiation, our results serve as an explanation for the excellent agreement of experimental findings and earlier DFT calculations of defect-free crystals. However, Ti_1−*x*_Al*_x_*N seems to be a very fortunate case, since in other material systems the impact of vacancies on the phase stability can be much stronger and therefore has to be taken into account. Finally, we have evidenced that, although having negligible impact on the cubic to wurtzite transition, vacancies clearly affect the physical properties and result in a decrease of Young's modulus, bulk modulus and shear modulus of cubic-structured Ti_1−*x*_Al*_x_*N.
